# Supplementary material for: An effector protein of the wheat stripe rust fungus targets chloroplasts and suppresses chloroplast function
Source: Nat Commun. 2019 Dec 5;10:5571. doi: 10.1038/s41467-019-13487-6 (PMC6895047; doi:10.1038/s41467-019-13487-6)
Supplement: Supplementary file 1 — Supplementary Information [file 41467_2019_13487_MOESM1_ESM.pdf]

An effector protein of the wheat stripe rust fungus targets chloroplasts and suppresses chloroplast function

Xu et al.

Supplementary Fig. 1

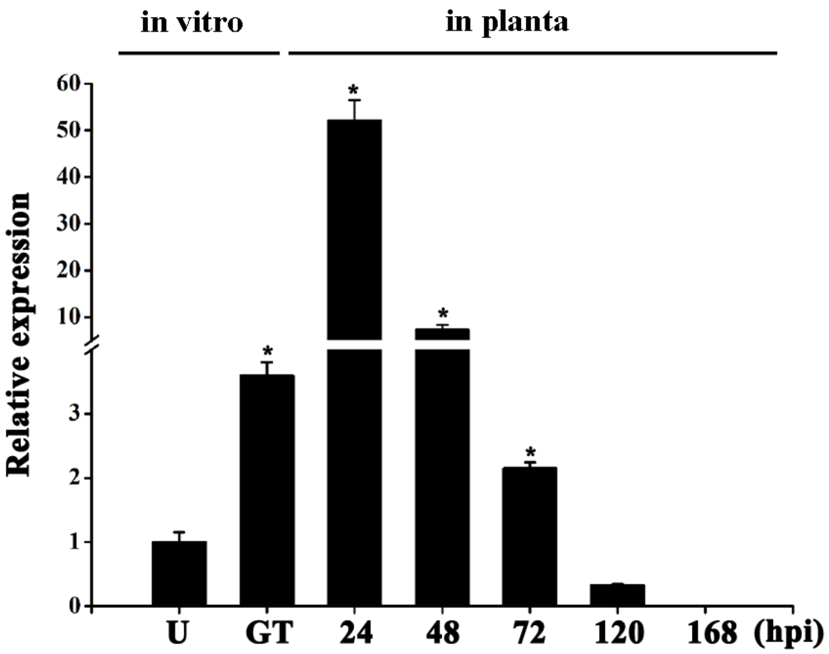

Supplementary Fig. 1 Expression pattern of *Pst_12806* is determined by qRT-PCR. The expression level of *Pst_12806* was assayed with RNA isolated from urediniospores (U), germ tubes (GT), and leaves of wheat cultivar Suwon 11 inoculated with urediniospores of *Pst* CYR31 sampled at 24, 48, 72, 120 and168 hpi (hour post inoculation). Relative expression was calculated by the comparative $2^{-\Delta\Delta Ct}$ method. Standard deviation and the mean fold changes_were calculated with results from three independent biological replications. The asterisk (*) indicates the significant difference compared to urediniospores (U) (*P* <0.05, Unpaired two-tailed Student's *t*-test).

Supplementary Fig. 2

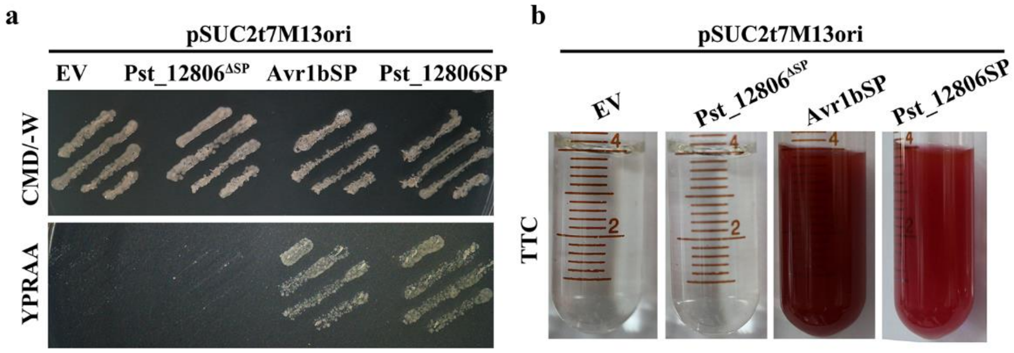

Supplementary Fig.2 Functional evaluation of the signal peptide of Pst_12806. a The function of signal peptides of Pst_12806 (Pst_12806SP) was identified using the yeast *Saccharomyces cerevisiae* (yeast YTK12 strain) cells containing the pSuc2t7M13ori vector, Avr1b, Pst_12806SP or Pst_12806$^{\Delta SP}$. The yeast transformants were cultured on CMD-W medium (yeast grew well without invertase secretion) and YPRAA medium (yeast only grew with invertase secretion)， respectively. **b** The enzymatic activity of invertase was detected by the reduction of 2, 3, 5-Triphenyltetrazolium Chloride (TTC) to insoluble red colored 1, 3, 5-Triphenylformazan (TPF). The transformed cells were collected in tubes to detect the enzymatic activity of invertase.

Supplementary Fig. 3

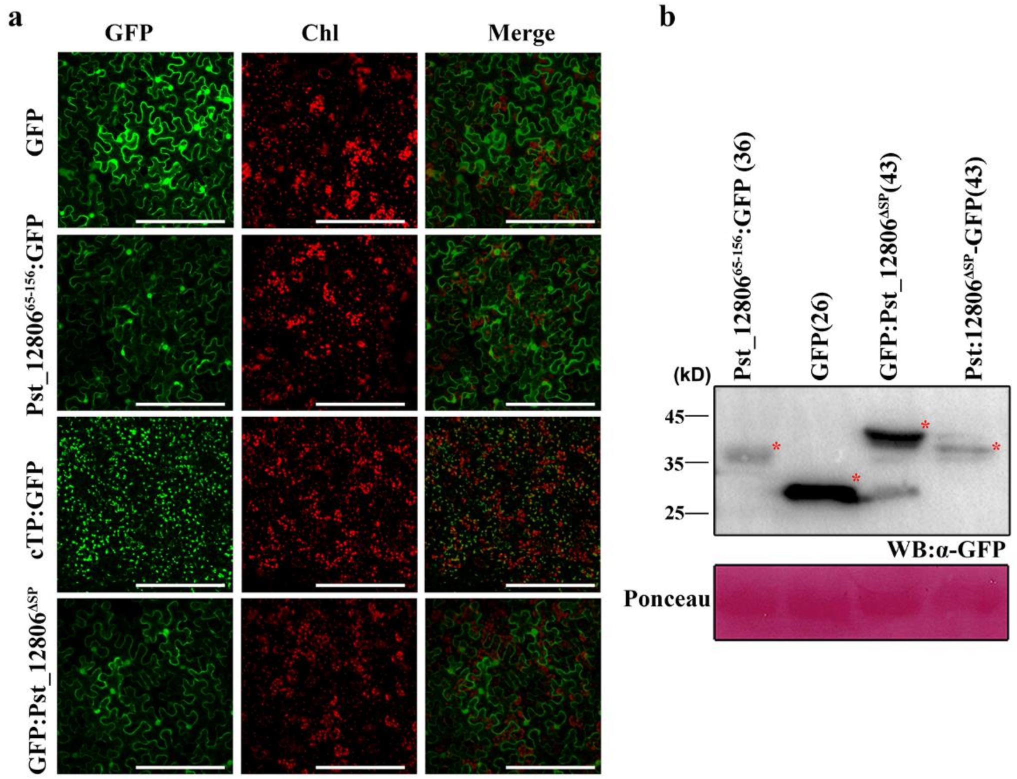

Supplementary Fig. 3 The cleave of the cTP of Pst_12806 in plant cells and its importance for chloroplast localization. a Leaves of *N. benthamiana* infiltrated with *Agrobacterium* cells expressing Pst_12806$^{65\text{-}156}$:GFP, cTP:GFP, GFP: Pst_12806$^{\Delta SP}$ and GFP constructs were examined by epifluorescence microscopy at 48 h to determine its function for chloroplast localization. Pst_12806$^{65\text{-}156}$:GFP has no signal peptide and chloroplast transmit (cTP), Chl, chlorophyll. Bar=200 μm. **b** The size of corresponding proteins (Pst_12806$^{65\text{-}156}$:GFP, Pst_12806$^{\Delta SP}$:GFP, GFP:Pst_12806$^{\Delta SP}$) was detected with anti-GFP by western blot analysis. The predicted molecular weight of these proteins is shown in the brackets and asterisks are represented the actual bands of these proteins. Ponceau staining of rubisco was performed as an internal reference protein.

Supplementary Fig. 4

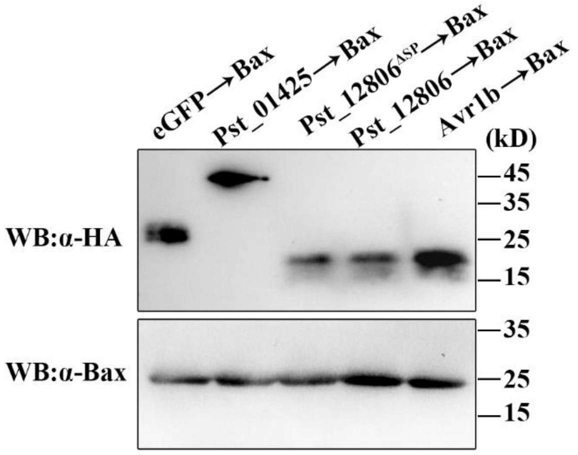

Supplementary Fig. 4 Proteins were detected by western blot analysis in the suppression of Bax-triggered cell death assay. Bax and eGFP proteins were extracted from *N. benthamiana* leaves after 60 h infiltrated into *Agrobacterium* cells carrying Bax. Pst_12806:HA, Pst_12806$^{\Delta SP}$:HA, Avr1b:HA, Pst_01425:HA and eGFP:HA proteins were detected by western blot with anti-HA antibody. Bax protein was detected by western blot with anti-Bax antibody.

Supplementary Fig. 5

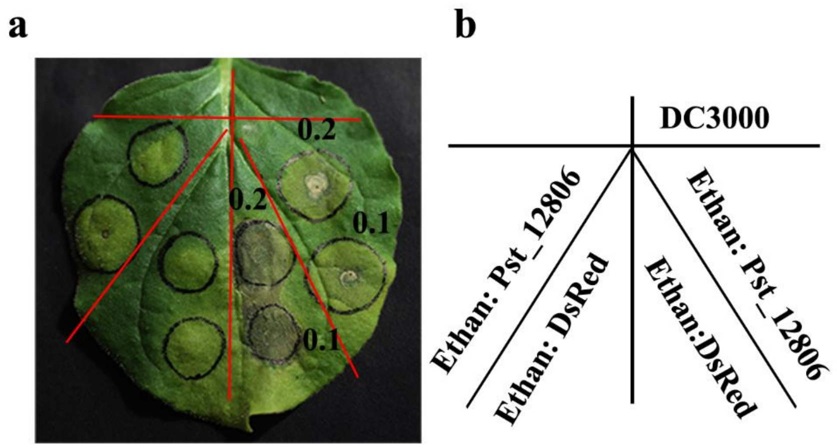

Supplementary Fig. 5 Pst_12806 delays HR induced by DC3000 on leaves of *N. benthamiana*. a Phenotype of HR suppression by Pst_12806. Ethan cells with the labeled Pst_12806 or DsRed were infiltrated into 4-weeks-old *N. benthamiana* leaves with an OD_{600} of 1.0. The DC3000 cells with an OD_{600} of 0.2 or 0.1 were infiltrated at the same sites after 24 h of Ethan strains infiltration. Representative images were captured at 5 day after DC3000 strain infiltration. The numbers on the picture are the value of OD_{600} of DC3000 strain **b** Schematic of infiltration, Ethan cells expressing the labelled Pst_12806 or DsRed were used for infiltration assays.

Supplementary Fig. 6

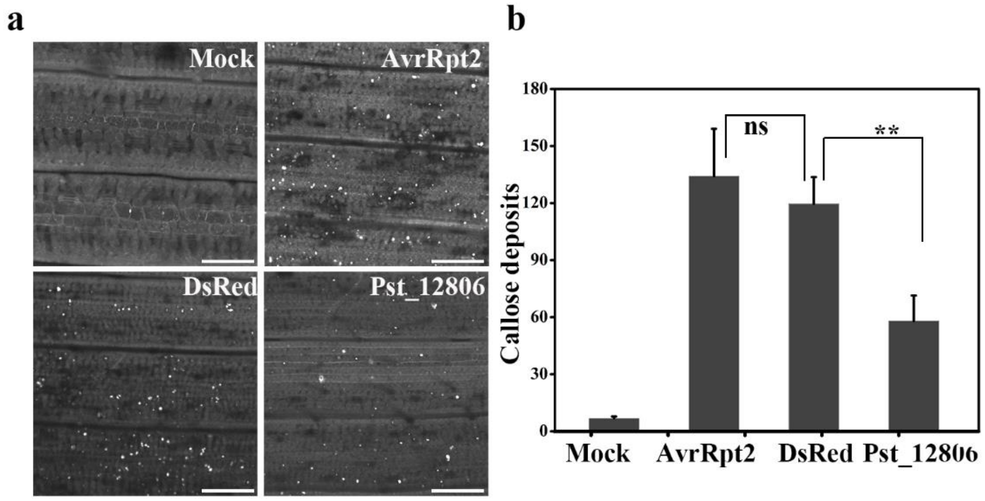

Supplementary Fig. 6 Ethan cells with the labeled Pst_12806 inhibits callose deposits on wheat leaves. a Wheat samples were extracted at 24 h on the second leaf of the cultivar Suwon 11 after Ethan cells infiltration and stained with 0.05% aniline blue overnight. Ethan cells with the labeled DsRed were negative controls and bacteria cells expressing AvrRpt2 induced callose deposits on wheat leaves. Mock was treated with distilled water instead of Ethan cells infiltration. Representative images were extracted using CellSens Entry software. Bar=200 μm. **b** Pst_12806$^{\Delta SP}$ suppresses callose deposits induced by bacteria in wheat leaves. Ethan carrying AvrRpt2 induced callose deposits in wheat leaves. The average and SD were derived from thirty 1-mm^2 areas in three biological replicates. * indicates significant differences in callose deposition when compared to that in wheat leaves treated with *Ethan* carrying DsRed (**, *P*<0.01; ns, not significant; Unpaired two-tailed Student's *t*-test).

Supplementary Fig. 7

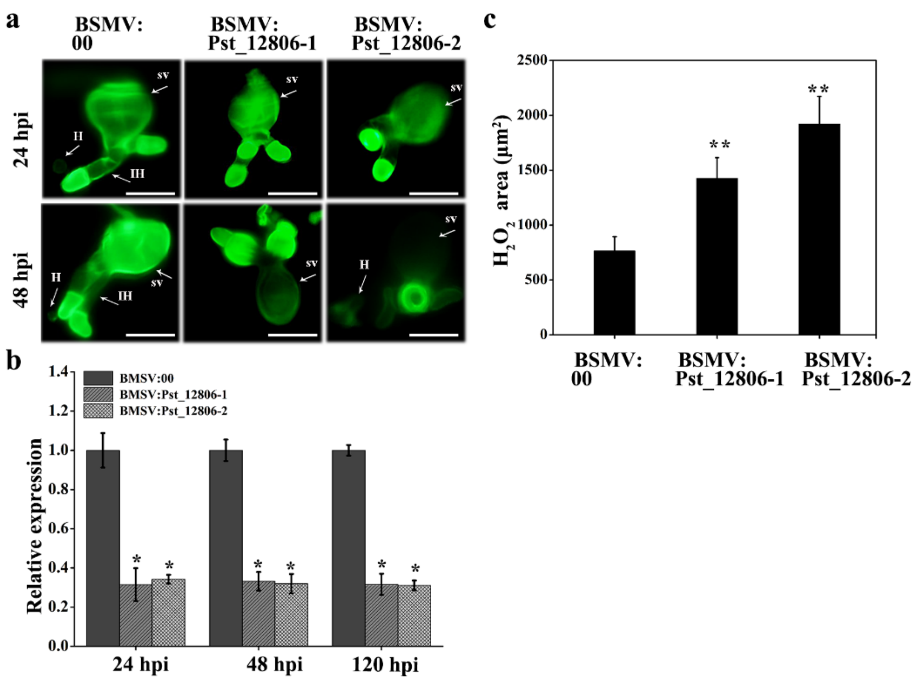

Supplementary Fig. 7 Silencing of *Pst_12806* by HIGS decreases the development of *Pst*. a Representative image from the *Pst_12806* knockdown wheat plants were obtained using CellSens Entry software. The wheat leaves infected by BSMV:Pst_12806 and BSMV:00 were inoculated with *Pst* CYR31 on the 4th leaves as Fig.4c as described and harvested at 24 hpi and 48 hpi (hour post inoculation with *Pst*). *Pst* hyphae were stained by WGA and then observed using an Olympus BX-51 microscope (ocular: 10×; objective: 20×). IH, infectious hyphae; H, haustorium; Bar= 50 μm. **b** The silencing efficiency in Pst_12806 knockdown wheat leaves was assessed using qRT-PCR. The samples were collected for RNA extraction as Figure 4d as described. The mean and standard deviation were calculated from three independent biological replications. **c** H_2O_2 areas were assessed using the CellSens Entry software in the same wheat samples as described above at 120 hpi. The means and standard deviation were analyzed from 50 infection sites of three independent biological repeats. The asterisk (*) indicates the significant difference compared to the BSMV:00 treated plants (*, P <0.05; **, P <0.01, Unpaired two-tailed Student's *t*-test).

Supplementary Fig. 8

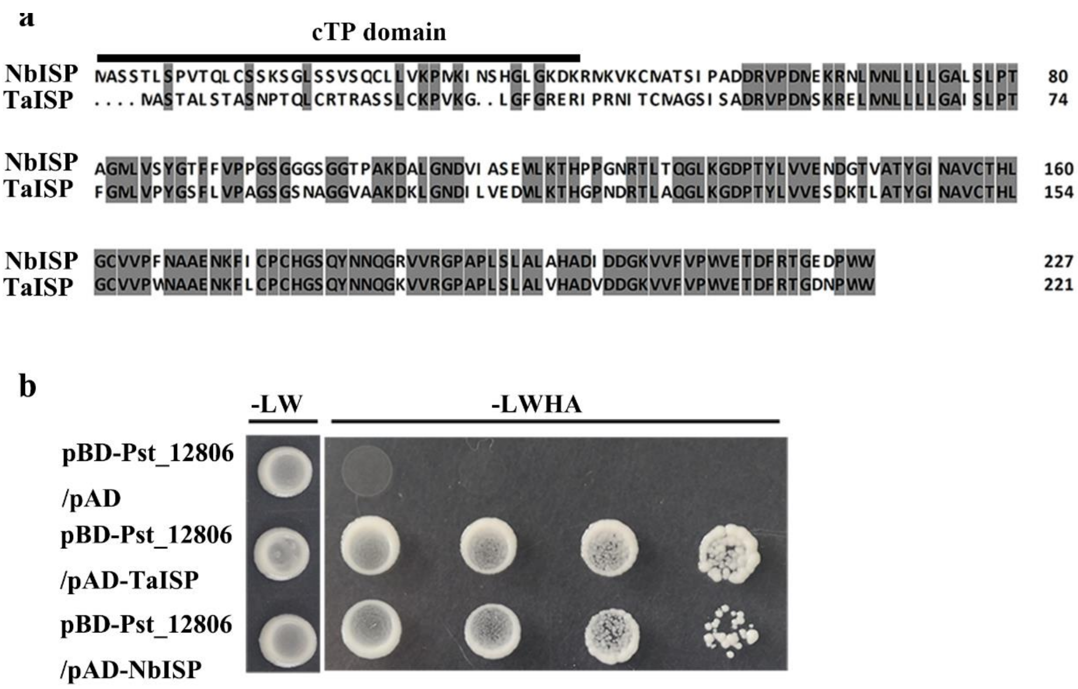

Supplementary Fig. 8 Pst_12806 interacts with NbISP in the Y2H assay. a Alignment of the peptide sequences of NbISP and TaISP. The sequences of *NbISP* and *TaISP* were amplified from the cDNA of *N. benthamiana* the and wheat cultivar Suwon 11, respectively. The sequence alignment was generated using DNAMAN software (version 6). The position of the cTP domain is indicated with a black line. The similarity of peptide sequences was shaded with black region. **b** The interaction between Pst_12806 and NbISP of *N. benthamiana* was confirmed in the Y2H assay. Yeast cells of AH109 strain transformed with the labeled constructs were assayed and were captured at 5 days after yeast cells with labelled proteins grown on SD medium lacking LW or LWHA. –LW, lacking leucine and tryptophan. –LWHA, lacking leucine, tryptophan, histidine and adenine.

Supplementary Fig. 9

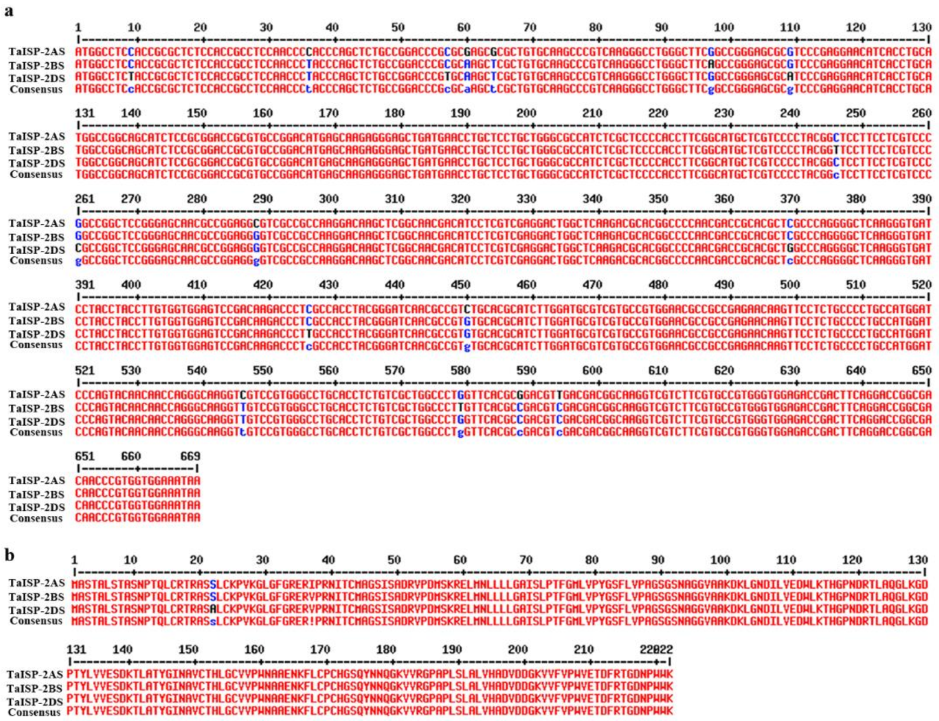

Supplementary Fig. 9 Three homologs of *TaISP* are of high similarity. a Nucleotide sequence alignments and **b** Amino acid sequence alignments. The sequences of three homologs of *TaISP* genes were obtained from the Ensembl plant database (http://plants.ensembl.org/index.html). Three homologs are located on chromosomes 2A, 2B and 2D, respectively and have several base variations marked with blue. The sequence alignment was generated at http://multalin.toulouse.inra.fr/multalin/multalin.html.

Supplementary Fig. 10

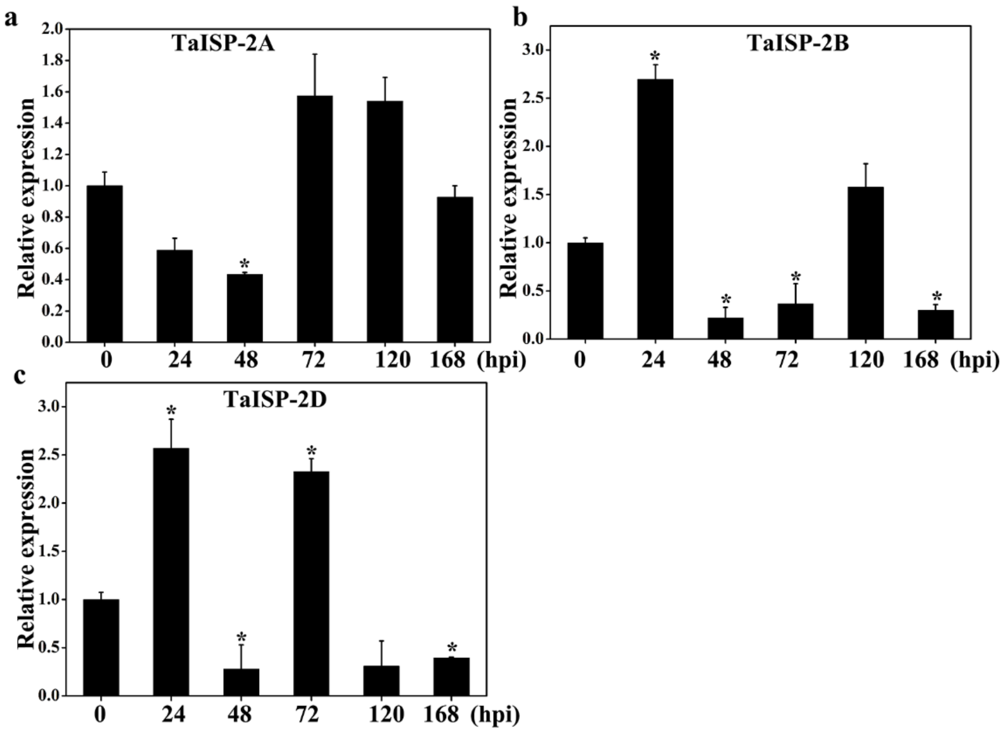

Supplementary Fig. 10 *TaISP* genes on chromosomes 2B and 2D are significantly induced by the *Pst* CYR31 in the Suwon 11 cultivar. a-c Transcript levels of *TaISP* on chromosomes 2A, 2B and 2D, respectively. The *TaISP* transcript levels of three copies was evaluated using qRT-PCR in wheat infected by *Pst* CYR31 at 0, 24, 48, 72 120 and 168 hpi (hour post inoculation with *Pst*), respectively. Relative expression was calculated by the comparative $2^{-\Delta\Delta Ct}$ method. Standard deviation and the mean fold changes were calculated with results from three independent biological replications. The asterisk (*) indicates the significant difference compared to the sample at 0 hpi (P <0.05, Unpaired two-tailed Student's t-test).

Supplementary Fig. 11

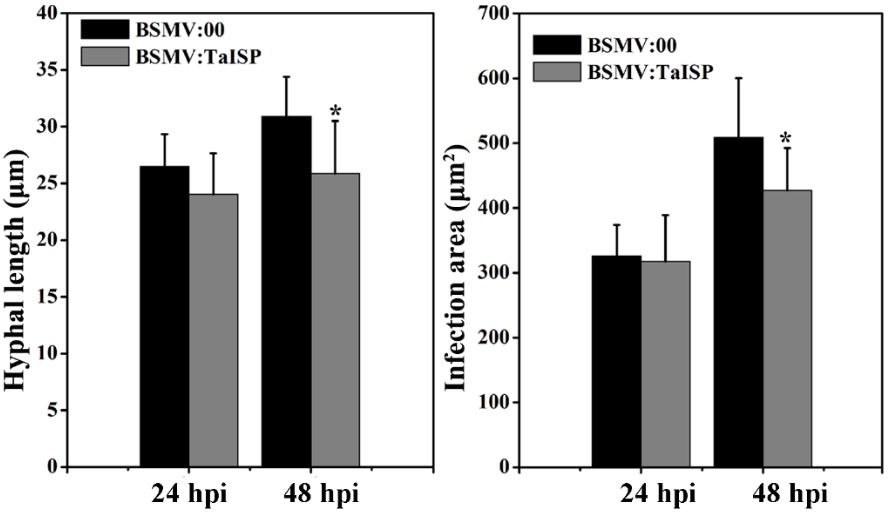

Supplementary Fig. 11 Hyphal length and infection areas decrease in *TaISP* knockdown wheat plants. *TaISP* knockdown plants were inoculated with *Pst* CYR31 on the 4th leaves, and sampled at 24 hpi and 48 hpi (hour post inoculation with *Pst*). Length and infection areas of *Pst* were analyzed using CellSens Entry software from 50 infection sites of each biological replicates. *Pst* hyphae were stained by WGA and then observed using an Olympus BX-51 microscope (ocular: 10×; objective: 20×), and the data were assessed using CellSens Entry software. The mean and standard deviation were calculated from three independent biological replications. The asterisk (*) indicates the significant difference compared to the BSMV:00 treated plants (P <0.05, Unpaired two-tailed Student's t-test).

Supplementary Fig. 12

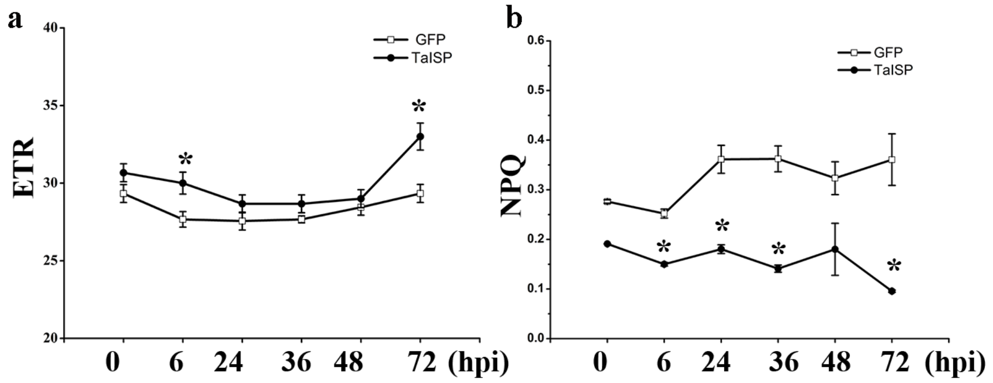

Supplementary Fig. 12 TaISP regulates plant photosynthesis in *N. benthamiana.* TaISP or GFP overexpressed in *N. benthamiana* via *Agrobacterium* transformation. Closed squares represent GFP and closed circles represent TaISP. **a** ETR (electron transport rate) was evaluated in TaISP overexpression plants at 0, 6, 24, 36 48, 72 hpi (hour post inoculation with *Agrobacterium*). **b** The value of NPQ (nonphotochemical quenching) was assayed in TaISP overexpression plants at 0, 6, 24, 36 48, 72 hpi. The data were measured using a PAM-2500 instrument under 2000 LUX light. The mean and standard deviation were calculated from three independent biological replications. The asterisk (*) indicates the significant difference compared to GFP treated plants (P <0.05, Unpaired two-tailed Student's *t*-test).
