# Supplementary material for: An effector protein of the wheat stripe rust fungus targets chloroplasts and suppresses chloroplast function
Source: Nat Commun. 2019 Dec 5;10:5571. doi: 10.1038/s41467-019-13487-6 (PMC6895047; doi:10.1038/s41467-019-13487-6)
Supplement: Supplementary file 2 — Description of Additional Supplementary Files [file 41467_2019_13487_MOESM2_ESM.pdf]

Description of Additional Supplementary Files

File Name: Supplementary Data 1
Description: Brief description of potential targets of Pst_12806 using the Y2H system

File Name: Supplementary Data 2
Description: Primers used in this study
