# Supplementary material for: An effector protein of the wheat stripe rust fungus targets chloroplasts and suppresses chloroplast function
Source: Nat Commun. 2019 Dec 5;10:5571. doi: 10.1038/s41467-019-13487-6 (PMC6895047; doi:10.1038/s41467-019-13487-6)
Supplement: Supplementary file 3 — Supplementary Data 1 [file 41467_2019_13487_MOESM3_ESM.pdf]

Supplementary Data 1 Brief description of potential targets of Pst_12806 using the Y2H system

Times	**Candidate targets**	**Gene bank**	**Species**
9	chloroplast fructose-1,6-biphosphate aldolase	A0A3B6RIU4	Triticum aestivum
4	unnamed protein product (DUF538 domain)	CDM86991	Triticum aestivum
3	isocitrate lyase	AKG97058	Triticum aestivum
2	E3 ubiquitin-protein ligase SINA-like 6	CDM80861	Triticum aestivum
2	homeobox-leucine zipper protein ROC9	CDM84292	Triticum aestivum
2	putative Rieske Fe-S precursor (TaISP)	AAM88439	Triticum aestivum
1	40S ribosomal protein S28	CDM87113	Triticum aestivum
1	bZIP transcription factor family protein	CAW48329	Triticum aestivum
1	Shaggy-like kinase	AGK41248	Triticum aestivum
1	superoxide dismutase (mitochondrion)	ACO90195	Triticum aestivum
1	Phosphoribulokinase, chloroplastic	CAA41020	Triticum aestivum
1	ankyrin-1-like isoform X1	CDM81963	Triticum aestivum
1	ribulose-1,5-bisphosphate carboxylase/oxygenase small subunit	BAB19815	Triticum aestivum
1	chloroplast inositol phosphatase-like protein	AAU82110	Triticum aestivum
1	serine/threonine-protein phosphatase 6	CDM82750	Triticum aestivum
1	geranylgeranyl hydrogenase	AAZ67145.2	Triticum aestivum
1	eukaryotic transcription factor NF-Y subunit C	AJS09958	Triticum aestivum
1	transcription factor TGAL1	CAW48333	Triticum aestivum
2	Elongation factor 2	EMS67172	Triticum urartu
1	Glycine dehydrogenase [decarboxylating] B, mitochondrial	EMS46370	Triticum urartu
1	NAC domain-containing protein 7	EMS66030	Triticum urartu
1	Lactation elevated protein 1	EMS61152	Triticum urartu
1	putative calcium-binding protein CML27	EMS63062	Triticum urartu
1	protein ACCELERATED CELL DEATH 6-like	EMS35691	Triticum urartu
1	MFS transporter	EMS62968	Triticum urartu
2	60S ribosomal protein L10-2	XP_020147355	Aegilops tauschii subsp. Tauschii
1	TPR repeat-containing protein ZIP4	XP_020157705	Aegilops tauschii subsp. Tauschii
1	Phototropin-2	XP_020176399	Aegilops tauschii subsp. Tauschii
1	hypothetical protein F775_44012 (HAD_like domain)	AER62215	Aegilops tauschii subsp. Tauschii
1	flowering locus K homology domain-like	XP_020160073	Aegilops tauschii subsp. Tauschii
1	Myb family transcription factor APL	XP_020150564	Aegilops tauschii subsp. Tauschii
1	ankyrin repeat-containing protein	BAK03736	Hordeum vulgare subsp. vulgare
1	protein scribble homolog isoform X4	BAJ99674	Hordeum vulgare subsp. vulgare
4	2-oxoisovalerate dehydrogenase subunit beta 1, mitochondrial	XP_003557527	Brachypodium distachyon
2	HAUS augmin-like complex subunit 5	XP_003578074	Brachypodium distachyon
1	GDSL esterase/lipase At2g04570-like	XP_024317383	Brachypodium distachyon
1	mitochondrial import receptor subunit TOM6 homolog	KQK14113	Brachypodium distachyon
1	heterogeneous nuclear ribonucleoprotein 1	PKU87734	Brachypodium distachyon
1	protein LIKE COV 1	XP_003568050	Brachypodium distachyon
1	Cysteinyl-tRNA synthetase	Q0IZQ2	Oryza sativa Japonica Group
2	hypothetical protein PSTG_13411	KNE93237	Puccinia striiformis f. sp. tritici PST-78
1	hypothetical protein PSTG_06809	KNE99957	Puccinia striiformis f. sp. tritici PST-78
