# Supplementary material for: An effector protein of the wheat stripe rust fungus targets chloroplasts and suppresses chloroplast function
Source: Nat Commun. 2019 Dec 5;10:5571. doi: 10.1038/s41467-019-13487-6 (PMC6895047; doi:10.1038/s41467-019-13487-6)
Supplement: Supplementary file 4 — Supplementary Data 2 [file 41467_2019_13487_MOESM4_ESM.pdf]

Supplementary Data 2 Primers used in this study

Name	sequence(5'-3')	application
HASP268-F	gGAATTCGGGGCGATCAGGGCT	
HASP268-R	cgGGATCCTCAATGAGCTTGGACGAGC	
TaISP-F	GGAATTCCATATGATGGCCTCTACCGCGC	
TaISP-N-F	GGAATTCCATATGATGGCCTCTACCGCGCTCTCCA	
TaISP-N-R	ccgCTCGAGTTAGCCGGCGGGGACGAGGA	
TaISP-C-F	GGAATTCCATATGAAGACGCACGGCCCCAACG	
TaISP-C-R	ccgCTCGAGTTATTTCCACCACGGGTTGTCGC	
TaISP-R	ccgCTCGAGTTATTTCCACCACGGGTTGT	Interaction assay
TaISP-PCH-F	TTACAATTATCGATACAATGATGGCCTCTACCGCGCTC	
TaISP-PCH-R	CTCATTAAAGCAGGACAAGCAGCGTAGTCTGGGACGTCGTATGGGTATTTCCACCACGGGTTGTCG	
Pst_12806$^{65-156}$-F	gGAATTCGGGGCGATCAGGGCT	
Pst_12806$^{65-156}$-R	cgGGATCCcATGAGCTTGGACGAGC	
TaISP-F	gGAATTCATGGCCTCTACCGCGCTC	
TaISP-R	cgcccgggTTTCCACCACGGGTTGTCG	
cTP-YFP-F	gGAATTC ATGCGATCAGGGCTGTTGCG	
cTP-YFP-R	cgGGATCCcGGTCTCGATCATAACCCGGTGA	
cTP-F	cccatggATGCGATCAGGGCTGTTGCG	
cTP-R	cgactagtGGTCTCGATCATAACCCGGTGA	
Pst_12806$^{65-156}$-F	cccatggATATGCATCCGCTTCTCCAAC	
Pst_12806$^{65-156}$-R	cgactagtATGAGCTTGGACGAGCACC	
Pst_12806-F	cccatggATATGGGGGCGATCAGGGCT	Sublocalization
Pst_12806-R	cgactagtATGAGCTTGGACGAGCACCG	
GFP-Pst_12806$^{24-156}$-F	GGGGACAAGTTTGTACAAAAAAGCAGGCTTC ATGGCGATCAGGGCTGTTG	
GFP-Pst_12806$^{24-156}$-R	GGGGACCACTTTGTACAAGAAAGCTGGGTCATGAGCTTGGACGAGCACCG	
CTP1-CFP-S	ccatggCCATGGACTAGTATGCAGGGTCGAGCAATCT	
CTP1-CFP-AS	GCCCTTGCTCACCATCAAAGGACAATTTGGTCCGTTTC	
Pst_12806-F	gtacccgggATGGCCCGCCACCTC	
Pst_12806-R	ataagaatgcggccgcAGCGTAGTCTGGGACGTCGTATGGGTAATGAGCTTGGACGAGCACC	
Pst_12806$^{24-156}$-F	ggcatcgatATGGCGATCAGGGCTGTT	
Pst_12806$^{24-156}$-R	catgtcgacAGCGTAGTCTGGGACGTCGTATGGGTAATGAGCTTGGACGAGCACC	
Avr1b-F	gtacccgggACTGAGTACTCCGACGAAACC	Supression of PCD
Avr1b-R	ataagaatgcggccgcGCTCTGATACAGGTGAAAG	
BAX-F	gtacccgggATGGACGGGTCCGGG	
BAX-R	ataagaatgcggccgcGCCCATCTTCTTCCAG	
eGFP–F	gtacccgggATGGTAGATCTGACTAGTCCTAGG	
eGFP-R	ataagaatgcggccgcCTTGTACAGCTCGTCCAT	
Pst_12806-GY-F	GGGGACAAGTTTGTACAAAAAAGCAGGCTTCGCGATCAGGGCTGTTG	Supression of PTI
Pst_12806-GY-R	GGGGACCACTTTGTACAAGAAAGCTGGGTCTCAATGAGCTTGGACGAG	
Pst_12806-vigs-F1	ataTTAATTAATACTTCAGTCTGTCTTGCTCGC	
Pst_12806-vigs-R1	tatGCGGCCGCTGACTAAGTTGGAGAAGCGGAT	
Pst_12806-vigs-F2	ataTTAATTAACCTGACCCGGAACCAAA	Silencing assay
Pst_12806-vigs-R2	tatGCGGCCGCCGAATACGCCACCGGAG	
TaISP-vigs-F	ataTTAATTAACTCCACCGCCTCCAACC	
TaISP-vigs-R	tatGCGGCCGCCGAGCATGCCGAAGGTG	
Pst_12806-qRT-F2	ACTCCTACTTCAGTCTGTCTTGCTC	
Pst_12806-qRT-R2	TGCTATTAGTCTTAGTGGCCTTTG	
Pst_12806-qRT-F1	CTTCAGTCTGTCTTGCTCGC	
Pst_12806-qRT-R1	GGGGTCGGCTTTGGTT	
PstEF1-F	TTCGCCGTCCGTGATATGAGACAA	
PstEF1-R	ATGCGTATCATGGTGGTGGAGTGA	
TaEF-F	TGGTGTCATCAAGCCTGGTATGGT	
TaEF-R	ACTCATGGTGCATCTCAACGGACT	

NbAct-F	GTTGCTATACAAGCTGTTCTCTCG	
NbAct-R	GTCAAGACGAAGAATGACATGTGG	
NbPR1a-F	CGACCAGGTAGCAGCCTATG	
NbPR1a-R	TCTCAACAGCCTTAGCAGCC	
NbPR2-F	GGGCTGTTAATTTGCAGTATCC	
NbPR2-R	GGTTTATAACATCTTGGTCTGATGG	
NbWRKY12-F	CTCATCAGCTAGTTCATTTGATGC	qRT-PCR
NbWRKY12-R	AGCTCGGTCTTTGTTCTAAAAGC	
TaPR1-F	GAGAATGCAGACGCCCAAGC	
TaPR1-R	CTGGAGCTTGCAGTCGTTGATC	
TaPR2-F	AGGATGTTGCTTCCATGTTTGCCG	
TaPR2-R	AAGTAGATGCGCATGCCGTTGATG	
TaPR5-F	CAAGCAGTGGTATCAACGCAGAG	
TaPR5-R	GTGAAGCCACAGTTGTTCTTGATGTT	
TaISP-2A-F	GGCCTTACTGGTTTCGTAAATC	
TaISP-2A-R	GATGAGGCGCGGGTTATAT	
TaISP-2B-F	CAGTCCTAAGAACGCTGCTATA	
TaISP-2B-R	GTCGTCTACATCGTAGTACCAG	
TaISP-2D-F	ACGCTGCTACATAAGACTACTC	
TaISP-2D-R	AGATTCCGAGTCCTCAAAATGT	
Pst_12806SP-F	CGGAATTTTAATTAAGAATTCATGGCCCGCCACCTCAAACT	
Pst_12806SP-R	CACTATAGGGAGAACCTCGAGCCCCTCGACATCCTTCCAAGTTGT	
Pst_12806$^{24\text{-}156}$-F	CGGAATTTTAATTAAGAATTC ATGGCGATCAGGGCTGTTGC	
Pst_12806$^{24\text{-}156}$-R	CACTATAGGGAGAACCTCGAGATGAGCTTGGACGAGCACC	Secretion assay
