# Supplementary material for: An effector protein of the wheat stripe rust fungus targets chloroplasts and suppresses chloroplast function
Source: Nat Commun. 2019 Dec 5;10:5571. doi: 10.1038/s41467-019-13487-6 (PMC6895047; doi:10.1038/s41467-019-13487-6)
Supplement: Supplementary file 6 — Reporting Summary [file 41467_2019_13487_MOESM6_ESM.pdf]

nature research

Corresponding author(s): Xiaojie Wang

Last updated by author(s): Oct 18, 2019

Reporting Summary

Nature Research wishes to improve the reproducibility of the work that we publish. This form provides structure for consistency and transparency in reporting. For further information on Nature Research policies, see Authors & Referees and the Editorial Policy Checklist.

Statistics

For all statistical analyses, confirm that the following items are present in the figure legend, table legend, main text, or Methods section.

n/a	Confirmed	
☐	☒	The exact sample size (*n*) for each experimental group/condition, given as a discrete number and unit of measurement
☐	☒	A statement on whether measurements were taken from distinct samples or whether the same sample was measured repeatedly
☐	☒	The statistical test(s) used AND whether they are one- or two-sided<br>*Only common tests should be described solely by name; describe more complex techniques in the Methods section.*
☐	☒	A description of all covariates tested
☐	☒	A description of any assumptions or corrections, such as tests of normality and adjustment for multiple comparisons
☐	☒	A full description of the statistical parameters including central tendency (e.g. means) or other basic estimates (e.g. regression coefficient) AND variation (e.g. standard deviation) or associated estimates of uncertainty (e.g. confidence intervals)
☐	☒	For null hypothesis testing, the test statistic (e.g. *F*, *t*, *r*) with confidence intervals, effect sizes, degrees of freedom and *P* value noted<br>*Give P values as exact values whenever suitable.*
☒	☐	For Bayesian analysis, information on the choice of priors and Markov chain Monte Carlo settings
☐	☒	For hierarchical and complex designs, identification of the appropriate level for tests and full reporting of outcomes
☐	☒	Estimates of effect sizes (e.g. Cohen's *d*, Pearson's *r*), indicating how they were calculated

Our web collection on statistics for biologists contains articles on many of the points above.

Software and code

Policy information about availability of computer code

Data collection	BIO-RAD CFX software(version: 3.1) for qRT-PCR, PAM-2500 instrument (Heinz Walz Company, Germany) for chlorophyll fluorescence assay; CellSens Entry software (version: V1.7) for infection areas;
Data analysis	ImageJ software (version: 1.60) for analysis of Callose deposits; CellSens Entry software (version: V1.7) for analysis of length of hyphae and infection area;SignalP 4.0 (http://www.cbs.dtu.dk/services/SignalP/) for predication of signal peptide;Compute pI/Mw tool (http://web.expasy.org/compute_pi/) for molecular size of protein; LOCALIZER (http://localizer.csiro.au/index.html) and WoLF PSORT II (https://www.genscript.com/wolf-psort.html) for the localization of the secreted protein; HMMER software (http://www.ebi.ac.uk/Tools/hmmer/) for Protein domains;

For manuscripts utilizing custom algorithms or software that are central to the research but not yet described in published literature, software must be made available to editors/reviewers. We strongly encourage code deposition in a community repository (e.g. GitHub). See the Nature Research guidelines for submitting code & software for further information.

Data

Policy information about availability of data

All manuscripts must include a data availability statement. This statement should provide the following information, where applicable:

- Accession codes, unique identifiers, or web links for publicly available datasets
- A list of figures that have associated raw data
- A description of any restrictions on data availability

Data supporting the findings of this work are available within the paper and its Supplementary Information files. GenBank accessions include AAM88439 for TaISP genes, XP_016485041 for NbISP gene, KNE93802 for Pst_12806. The authors declare that the other data supporting the findings of this study are available from the corresponding author upon request.

Field-specific reporting

Please select the one below that is the best fit for your research. If you are not sure, read the appropriate sections before making your selection.

☒ Life sciences ☐ Behavioural & social sciences ☐ Ecological, evolutionary & environmental sciences

For a reference copy of the document with all sections, see nature.com/documents/nr-reporting-summary-flat.pdf

Life sciences study design

All studies must disclose on these points even when the disclosure is negative.

Sample size	4-week-old N. benthamiana for transiently expression;the second leaves for inoculation of virus or Pst; Fifty infection sites on the three leaves were analyzed for hyphal length, haustorium count and infection area.
Data exclusions	No data were excluded from the analyses. all data were used in this study in the origin data file.
Replication	Six independent biological replications were performed for HIGS assay and three independent biological replication for other assays. all biological replications confirmed the similar results.
Randomization	Four tobacco (8-10 leaves) were randomly distributed into one group for cell death assay. 30-35 wheat leaves for infiltration of virus and 25-30 wheat leaves were randomly allocated for HIGS and VIGS assay. 8-10 leaves of tobacco and wheat were randomly distributed for detection of chlorophyll fluorescence and ETR.
Blinding	The investigators were blinded to group allocation during the experiment and analysis.

Reporting for specific materials, systems and methods

We require information from authors about some types of materials, experimental systems and methods used in many studies. Here, indicate whether each material, system or method listed is relevant to your study. If you are not sure if a list item applies to your research, read the appropriate section before selecting a response.

Materials & experimental systems

n/a	Involved in the study	
☐	☒	Antibodies
☒	☐	Eukaryotic cell lines
☒	☐	Palaeontology
☒	☐	Animals and other organisms
☒	☐	Human research participants
☒	☐	Clinical data

Methods

n/a	Involved in the study	
☒	☐	ChIP-seq
☒	☐	Flow cytometry
☒	☐	MRI-based neuroimaging

Antibodies

Antibodies used	rabbit anti-GFP, cat. no. 50430-2-AP, Proteintech Group ; mouse anti-GFP, cat. no. sc-9996, Santa Cruz Biotechnology ; mouse anti-HA, cat. no. H3663, Sigma-Aldrich ; a secondary goat anti-mouse IgG (whole molecule)-peroxidase-conjugate antibody, cat. no. sc-516102, Santa Cruz Biotechnology
Validation	abbit anti-GFP, cat. no. 50430-2-AP,Rabbit Polyclonal,WB, IP, IHC, IF, ELISA (https://www.ptgcn.com/Products/eGFP-Antibody-50430-2-AP.htm); mouse anti-GFP, cat. no. sc-9996,mouse IgG2a κ,WB, IP, IF, FCM, ELISA (https://www.scbt.com/zh/p/gfp-antibody-b-2/;jsessionid=fQjX0mynSTobDkEH0SXGedNmsEjegWcTGb7GoWJHk_hpyG838ReE!711723271);mouse anti-HA, cat. no. H3663,Monoclonal Anti-HA antibody produced in mouse,immunocytochemistry,immunoprecipitation,western blot (https://www.sigmaaldrich.com/catalog/product/sigma/h3663?lang=zh&region=CN)
